# We still don’t know that our children need vitamin D daily: a study of parents’ understanding of vitamin D requirements in children aged 0-2 years

**DOI:** 10.1186/s12889-019-7340-x

**Published:** 2019-08-15

**Authors:** Rhiannon Eleanor Day, Roxane Krishnarao, Pinki Sahota, Meaghan Sarah Christian

**Affiliations:** School of Clinical and Applied Sciences, Leeds Beckett University, CL615A, City Campus, Leeds, LS1 3HE UK

**Keywords:** Vitamin D intake, Infants and children, Qualitative and quantitative

## Abstract

**Background:**

Vitamin D deficiency has been highlighted as a serious public health problem in the United Kingdom. One in four toddlers are not achieving the recommended intake for their healthy development. This study uses quantitative and qualitative methods to explore parents’ perceptions, awareness and behaviours around vitamin D intake, and the acceptability of and factors affecting purchasing of food and drink fortified with Vitamin D in children aged 0–2 years old.

**Methods:**

One hundred and ninety-four parents completed an online questionnaire, advertised to parents with one child aged up to 2 years on popular social media websites. The majority of participants were mothers, White-British ethnic background, aged 25–44 years. Participants provided an email address if they wanted to be contacted about the focus groups. Recruitment posters advertising the focus groups were placed in community centres. Eighteen participated in 5 focus groups (13 parents), and 5 individual interviews. A thematic analysis methodology was applied.

**Results:**

Fifty-seven percent (*n* = 110) of parents reported receiving information about vitamin D during pregnancy and 52% (*n* = 100) after the birth of their child. Parents reported a low level of satisfaction with vitamin D information: many thought it was limited and recommendations on supplements were unclear.

Parents wanted more information about vitamin D requirements for their child (80%, *n* = 153 out of 192 respondents, 2 non-response), about vitamin D and breastfeeding (56%, *n* = 108) and vitamin D and pregnancy (49%, *n* = 94). The recommendations were for simpler, easier to read, with specific and clearer guidelines; delivered regularly during routine appointments, at timely stages throughout pregnancy and after the birth. 23% (*n* = 45, out of 194 respondents) of parents did not know why vitamin D is important for health. Only 26% (*n* = 49, out of 192 respondents) of parents reported giving their youngest child a vitamin D supplement on most days of the week. The majority of parents (interview/focus group) wanted more information about foods/drinks fortified with vitamin D.

**Conclusion:**

Parents were generally not aware of the importance of vitamin D, dietary requirements including supplementation and the availability of vitamin D fortified foods. Major improvements are required for the effective promotion of vitamin D information to parents.

**Electronic supplementary material:**

The online version of this article (10.1186/s12889-019-7340-x) contains supplementary material, which is available to authorized users.

## Background

Vitamin D deficiency (plasma 25-hydroxy vitamin D (25-OH-D) < 25 nmol/L) has been highlighted as a serious public health problem in the United Kingdom (UK) [1]. Vitamin D (25-OH-D) is primarily obtained through dermal synthesis of Ultraviolet B (UVB) sunlight. It can also be obtained through the diet, but few foods contain vitamin D naturally and fortification of foods in the UK is limited [2]. National Diet and Nutrition Survey (NDNS) data suggests that the current diets of toddlers provide close to or above the reference nutrient intake for all vitamins except for vitamin D, with one in four toddlers not achieving the recommended vitamin D intake levels crucial to their healthy development [3]. It also highlighted that average intakes of vitamin D from food sources in children aged up to 4 years old were less than a third of the recommended amount [4], and that uptake of supplements is low in breastfeeding mothers and children under five [5]. Children under five are therefore at an increased risk of vitamin D deficiency and suggests that the current supplementation strategy may not be fully effective [6].

Vitamin D is essential for regulating calcium metabolism and promotes intestinal calcium absorption [7, 8], and thus is essential for bone health. A low serum 25-OH-D concentration of less than 30 nmol/l has been associated with a reduction in bone mass density in children and adolescents [9, 10], increased risk of rickets and hypocalcaemic seizures in young children [1, 6, 11–13], increased risk of osteomalacia in young and middle aged adults, and osteoporosis and fractures in older adults [14]. Research conducted in the UK over the past few decades, has highlighted the increasing burden of rickets [15], and the increased susceptibility of the Black, Asian and Minority Ethnic groups. The British Paediatric Surveillance Unit survey reported a total of 91 cases of hypocalcaemic seizures due to vitamin D deficiency (85%, *n* = 77 were infants) from September 2008–2011. This equates to an annual incidence of 3.49 per million children (aged 0–15 years), with the South Asian population having the highest incidence (26.04 per million) [15]. This high incidence of rickets in the South Asian population, compared to the White population, precludes rickets from being classified as a rare disease [15]. Prolonged vitamin D deficiency could also have detrimental consequences to health in later life, for example vitamin D insufficiency has been associated with other health problems in adulthood, including cardiovascular disease, diabetes and autoimmune diseases [16, 17], which has implications for future health services treatment and management costs.

In June 2016, the Scientific Advisory Committee on Nutrition (SACN) [9], recommended that the Department of Health (DH) update their vitamin D guidelines. The recommendations are that all babies under 1 year should have a daily 8.5 to 10 microgram vitamin D supplement (including exclusively breastfed and partially breastfed infants, from birth), unless they are receiving more than 500ml of formula per day, as formula is already fortified, and a daily 10 microgram vitamin D supplement for children aged 1 up to 4 years [18]. Vitamin D supplementation of 10 micrograms per day for the entire population (from 5 years up) particularly in Winter (October–April), is also recommended. Public Health England (PHE) also advises that all women receive advice from early pregnancy about the benefits of taking vitamin D throughout pregnancy and lactation [19, 20]. It has been reported however, that these universal recommendations are not being followed by women and that this is contributing to health inequalities [21, 22]. The National Institute for Health and Care Excellence (NICE) [23] has made a number of recommendations to increase vitamin D supplement use; including increasing awareness of the importance of vitamin D, improving local availability and access to the Healthy Start scheme (which comprises targeted provision of free vitamin supplements for women throughout pregnancy and children (vitamin drops containing A, C, D for children aged 6 months to 5 years old), for all low income families)) [24]. However, uptake of these vitamin drops has been shown to be poor and as low as 1.5% in some areas with targeted distribution [25]. A report commissioned by the Department of Health’s Policy Research Programme [26], found a number of issues relating to poor uptake; such as parents find it difficult to access Healthy Start vitamins, health professionals do not promote the scheme and eligible families are often unaware of it. It also identified that the distribution system is complex, confused and weak and there is little motivation by mothers to either take vitamins themselves or give them to their children. The lack of awareness around the importance of vitamin D in parents is most concerning [25]. Research commissioned by the Vitamin D Mission suggests that 20% of parents of children under 5 years do not know that their child is at risk of insufficiency, and a third of parents have said that they have never received information about the need for vitamin D [25].

In order to effectively improve vitamin D intake by young children, there needs to be increased knowledge and awareness of the importance of vitamin D for infants and young children. There is little research exploring parents’ understanding and perceptions of vitamin D information, particularly in the UK [2], as well as preferred methods for increasing vitamin D intake in their children. There is thus a need to understand the level of awareness and knowledge of parents around vitamin D and evaluate the effectiveness of the methods used for promoting and educating about vitamin D. Therefore, the objectives of the current study were to explore parents’ perceptions, awareness and behaviours of vitamin D intake; to explore the acceptability of and factors affecting purchasing of food and drink fortified with vitamin D; and to propose strategies for increasing vitamin D intake during pregnancy, breastfeeding and in children aged 0–2 years. The results can be used to develop effective public health strategies to improve vitamin D status.

## Methods

A mixed methods design, involving both quantitative and qualitative research methods was used to meet the objectives of the study. Data collection, data management and data analysis was undertaken by the Nutrition and Childhood Obesity Research group at Leeds Beckett University.

### Online parent questionnaire

A questionnaire was developed for this study, for parents with a child aged up to 2 years old to explore:

- Vitamin D education/information received;
- Understanding and knowledge of vitamin D;
- Vitamin D in families’ diets.

The questionnaire was developed and piloted for completion online (only one questionnaire to be completed per family), with appropriate format and layout incorporated into the design with the aim to capture a national sample. The questionnaire was constructed using Snap Surveys Ltd. [Bristol, UK], an application that enables the compilation of questionnaires and collection of data. The first section of the questionnaire collected information about where parents retrieved advice on health and nutrition and information about vitamin D specifically; how (format) they received vitamin D information and their views on and recommendations for this information. The second section explored knowledge and understanding of the role of vitamin D in the body. The third section explored families’ intake of vitamin D including their use of fortified foods and drinks; factors that would encourage use of fortified foods and drinks and which products parents would be willing to buy. It also explored frequency of vitamin D supplement use and reasons for supplement use. The final section of the questionnaire obtained demographic and socioeconomic information and allowed parents to select whether they wanted to participate in a focus group. The questionnaire is included as a supplementary file (Additional file 1). Postcode was collected on the questionnaire, and from this information, the Index of Multiple Deprivation (IMD) was devised. The Index of Multiple Deprivation (IMD) developed by the Department of Communities and Local Government in England [27], provides an official relative measure of deprivation for small areas across England, using postcode data based on 7 domains (income deprivation, employment deprivation, education, skills and training deprivation, health deprivation and disability, crime, barriers to housing and services and living environment deprivation). The IMD ranks every neighbourhood in England from 1 (most deprived area) to 32,844 (least deprived area). Deprivation deciles are calculated by ranking the 32,844 neighbourhoods from most deprived to least deprived, by dividing them into 10 equal groups, ranging from most deprived 10% of neighbourhoods to the least deprived 10% of neighbourhoods (score 1–10) [27].

### Focus groups

The purpose of the focus groups with parents was to gain greater insight into awareness, knowledge and perceptions of the importance of vitamin D; including awareness of vitamin D recommendations (e.g. health visitor information/advice); supplementation and knowledge and acceptability of vitamin D fortified dietary sources was also explored, including facilitators and barriers to purchasing fortified foods/drinks. A semi-structured focus group schedule was developed based on the data gathered from the online questionnaire.

### Recruitment of participants

The on-line questionnaire allowed respondents to tick a box at the end of the questionnaire to express their interest in participating in a focus group. These respondents were contacted and asked to attend a focus group. Voluntary led community-based playgroups and community/family information centres across Leeds, north England, were also asked to display a recruitment poster for the focus groups. Parents who were interested in participating in the focus group were requested to contact the research team directly.

### Procedure

All focus group (and interview) participants were sent an information sheet, which explained the purpose of the study and the focus group process. Written informed consent to participate was obtained from all participants, prior to commencement of the focus group. Measures were adhered to with respect to data storage during the study, and participants were free to withdraw from the evaluation at any time. All focus groups/ interviews were digitally-recorded after written consent had been obtained from participants. Individuals involved in the evaluation were also assured that they would not be identifiable in the report of findings.

### Ethics

Ethical approval was provided by Leeds Beckett University ethics review committee (reference number 28507) and the National Health Service (NHS) ethics, via the Integrated Research Application System (IRAS) (application number 213906). Parents undertaking the online and paper-based questionnaire were made aware at the start of the questionnaire how the data would be used. To encourage participation a free prize draw of a £50 high street shopping voucher was offered. Parents wishing to be entered into the prize draw were asked to provide a contact email address. Participants were also offered an incentive to encourage participation in the focus groups. Parents participating in a focus group each received a £5 high street shopping voucher.

### Data analysis

#### Questionnaire analysis

Individual level data was exported from the survey software SNAP professional 11 Snap Surveys Ltd. [Bristol, UK], as a csv.file. Microsoft Excel was used to calculate basic descriptive statistics such as counts, means and percentages and to create tables. The data from the questionnaire was analysed using percentages, these are presented as whole numbers and/or to one decimal place, when required.

#### Qualitative analysis

The audio files of the interviews and focus groups were transcribed with the focus on the content and essence of the discussions. The transcripts of the interviews and focus groups were anonymised; participants’ names were replaced with unique identifiers and identifying details (e.g. names of individuals and places) referred to within transcripts have been replaced with pseudonyms. A thematic analysis methodology was adopted in evaluating the qualitative data [28]. The analysis was conducted over a number of stages. After all interview data had been transcribed verbatim, members of the evaluation team read and familiarised themselves with the content of the transcripts. Based on this, a coding framework was developed, which was derived from thematic areas of interest within the data itself. The coding framework was refined and agreed amongst the research team and applied to the original transcripts to extract major themes.

## Results

The link to the online questionnaire was advertised on websites such as Netmums, Mumsnet, Facebook, Twitter and on group pages on Facebook such as the Leeds National Childbirth Trust pages and local parent forums. It was accessible to participants from the 16th February 2017 until 30th April 2017. To increase diversity of the sample, paper questionnaires were also completed at voluntary led playgroups, a community centre and in the café of a popular family and children’s retail outlet in Leeds. Questionnaires were completed by parents of children aged up to 2 years. In total 194 questionnaires were completed.

The focus groups/interviews were carried out during May and June 2017. Five focus groups were organised initially at three different locations within the Leeds area (3 held within the café of a popular retail outlet with families in the city centre and 2 carried out during playgroups within churches in low-income areas of Leeds). The research team also advertised a further focus group at a community centre based within a more diverse ethnic population of Leeds, but no parents showed interest in attending. Other parents who wanted to contribute to the study but were not able to attend a focus group, took part in a telephone interview with a member of the research team. Focus groups/interviews lasted between 25 and 40 min in length. One parent who was not able to attend a focus group or interview, also contributed a written response to the interview questions by email. Eighteen parents provided qualitative data in total: 13 parents participated in the 5 focus groups, 4 parents took part in a telephone interview and 1 parent contributed a written interview response.

### Characteristics

Table 1 presents the demographic and socioeconomic characteristics of the questionnaire respondents and the focus groups/interview participants. The vast majority of questionnaire respondents were women aged 25 to 44 years. Overall, a large proportion of the sample (69.6%, *n* = 135) had a level 4 qualification e.g. Higher National Diploma (HND), Degree and Higher Degree. This is much greater than the Leeds average (34%) with a level 4 qualification or the national average (38%) [29]. 87.2% (*n* = 169) of the participants were born in the United Kingdom from White-British backgrounds, and 12.9% (*n* = 25) respondents were of non-White British ethnic background (e.g. Asian or Asian British: Pakistani, Indian, Chinese, Black or Black British: Caribbean, Mixed: White and Black Caribbean, Other, White and Asian, White and Black Caribbean background). This is similar to the Leeds average for White-British population (85%) and the national average (86%). 10.3% (*n* = 20) of the sample were from the 20% most deprived areas, compared to an average of 31% for the Leeds area and 20% nationally [29]. Similarly, the majority of focus group/interview participants were mothers and were of White-British ethnic background. Overall, most of the participants were aged between 25 and 44 years old and just over half were educated to Level 4 (e.g. Degree, HND).

**Table 1:** Characteristics of participants

	Questionnaire		Focus Group	
Characteristics	N	(%)	N	(%)
Gender				
Male	6	(3.1)	1	(5.6)
Female	186	(95.9)	17	(94.4)
Not recorded	2	(1.0)		
Age				
Under 25	8	(4.1)	1	(5.6)
25-34	113	(58.3)	7	(38.9)
35-44	69	(35.6)	6	(33.3)
Over 45	2	(1.0)	2	(11.1)
Not recorded	2	(1.0)	2	(11.1)
Ethnic background				
White - British (English, Welsh, Scottish, Northern Irish)	169	(87.2)	15	(83.2)
White - Other	5	(2.6)	1	(5.6)
White - Irish	1	(0.5)		
Asian or Asian British - Pakistani	7	(3.6)		
Asian or Asian British – Indian	4	(2.1)	1	(5.6)
Asian or Asian British - Chinese	1	(0.5)		
Asian or Asian British - Other	1	(0.5)		
Black or Black British – Caribbean	1	(0.5)		
Mixed- Other	1	(0.5)		
Mixed – White and Asian	1	(0.5)		
Mixed – White and Black Caribbean	2	(1.0)		
Other	1	(0.5)		
Not recorded			1	(5.6)
Highest qualification				
No qualifications	1	(0.5)	1	(5.6)
Level 1 e.g. few than 5 GSCE’s	1	(0.5)		
Level 2 e.g. 5 or more GCSE	12	(6.2)	2	(11.0)
Level 3e.g. 2 or more A levels	26	(13.4)	1	(5.6)
Level 4 e.g. HND, Degree	135	(69.6)	10	(55.6)
Other	9	(4.6)		
Not recorded	10	(5.2)	4	(22.2)
Deprivation quintile*				
1 - 20% most deprived	20	(10.3)		
2	25	(12.9)		
3	40	(20.6)		
4	43	(22.2)		
5 - 20% least deprived	36	(18.5)		
Not recorded	30	(15.5)		

*IMD - deprivation quintiles score neighbourhoods from 1 (the most deprived 20%) to 5 (the least deprived 20%)

### Access to health information

Parents were asked in the questionnaire *‘where do you generally go for information and advice on health and nutrition?* (Respondents could choose more than one option). The results revealed that 87% (*n* = 169, out of 194 respondents to the question) of parents most commonly sought information online via medical websites; 61% (*n* = 119) reported using parenting websites; 61% (*n* = 118) would consult a health professional about health and nutrition; 55% (*n* = 106) asked family and friends and 31% (*n* = 61) said they used leaflets or booklets.

### Access to vitamin D information

Parents were asked in the questionnaire *‘have you ever searched for information on vitamin D?*’ (Respondents could choose more than one option). The results revealed that 43% (*n* = 84, out of 194 respondents) reported that they had searched for information relating to vitamin D during pregnancy, over a third of the sample reported searching for information relating to vitamin D and breastfeeding (35%, *n* = 68) and 38% (*n* = 73) reported searching for vitamin D information relating to their child. 36% (*n* = 69) of parents said that they had not searched for information on Vitamin D.

Parents were asked in the questionnaire ‘*did you (or your partner) receive any information about vitamin D from any of the following sources?’* (Respondents could choose more than one option). Of the 194 parents who completed the question, 57% (*n* = 110) of parents reported receiving advice on vitamin D during pregnancy by their midwife or health visitor, and 52% (*n* = 100) reported receiving advice after the birth of their child by their midwife or health visitor. Only 6% (*n* = 11) of parents reported receiving advice about vitamin D from their General Practitioner ((GP), family doctor) during pregnancy and 10% (*n* = 19) reported receiving advice from their GP after birth. Only 6% (*n* = 11) of parents reported receiving advice about vitamin D from an Early Years Practitioner (EYP) after birth (1%, (*n* = 2) during pregnancy), and 5% (*n* = 9) from a breast-feeding support worker after birth (2%, (*n* = 3) during pregnancy). Other sources (during pregnancy and after birth) included: group/class run by healthcare professionals/ or children’s centre (5%, *n* = 10), group/class run by private company (5%, *n* = 9), and NHS email/text messaging service (5%, *n* = 10).

The qualitative findings revealed that for many parents, the information about vitamin D was limited, with some parents receiving either only one discussion or no advice at all and many parents reported not receiving supportive written information about vitamin D. Many parents had not been informed why they need to take a vitamin D supplement and the importance of vitamin D for their baby/child was not made clear. Furthermore, some reported that other sources of vitamin D e.g. foods/drinks or sunlight were not discussed by their midwife or health visitor.

*“I don’t remember being given any advice while pregnant or after birth. It wasn’t until much later when it was brought up in a group that I became aware” (parent, interview 5)*

*“It was a checklist, one of the many things, no real explanation of why or benefits. In that environment, you just say okay” (parent 1, focus group 1)*

#### Perceptions of vitamin D information/advice provided to parents

Parents participating in a focus group/interview (*N* = 18), were asked to give their views on the information they received about vitamin D during pregnancy, after the birth of their child and when breastfeeding or bottle feeding, where relevant. Many of the questionnaire respondents also spontaneously provided written views on the advice/information about vitamin D they received, when asked *‘how could the information you received about vitamin D be improved’*. These views have been summarised together.

Parents who completed the questionnaire reported quite a low level of satisfaction with the information on Vitamin D they had received, for this question a 5-point Likert scale was used (very good, good, OK, poor, and very poor, respondents ticked one option). Only 8.7% (*n* = 12, out of 138 respondents to the question) indicated that it was very good, 26.0% (*n* = 36) good, 46.3% (*n* = 64) OK, however 14.5% (*n* = 20) rated the information as poor and 4.5% (*n* = 6) very poor.

The qualitative findings revealed that for many parents, there was too much information given at once for it all to be digested effectively. Consequently, some parents expressed difficulty with trying to remember the vitamin D information amongst all the other information given about the birth. Others could not access information about vitamin D from a suitable health care practitioner. Furthermore, some perceived there to be a lack of reliable information, which was often conflicting from different sources. Some parents also perceived that the vitamin D information was not appealing and eye catching in appearance.

*“I do think when they give you the information, it is not drip fed to you, it is here is a load of information and a load of leaflets” “It was quite boring in appearance and looks like a medical document. It wasn’t the one out of the whole pack of information that I got that I was drawn to and had real importance” (parent, interview 4)*

### Parent recommendations for vitamin D information

When asked if they would like more information about vitamin D (respondents could choose more than one option), 80% (*n* = 153, out of 192 respondents to the question, 2 non response) of parents surveyed said they would have liked more information about vitamin D requirements for their child, 56% (*n* = 108) wanted more information on vitamin D and breastfeeding and 49% (*n* = 94) on vitamin D and pregnancy. Only 9% (*n* = 19) stated they did not want more information and 6% (*n* = 12) were unsure.

#### Content of vitamin D information

Parent views from both the questionnaire and focus groups/interviews, have been used to compile a list of questions that parents would like the vitamin D education to address. These are presented in Table 2. Parents mainly wanted information about the sources of vitamin D, the importance of vitamin D and risks of deficiency, vitamin D requirements for their child, during pregnancy and breastfeeding and how to access vitamin D supplements.

**Table 2:** What parents stated they wanted to know about vitamin D

*Sources of Vitamin D*	
• What are the main sources of vitamin D? • What are the main food sources of vitamin D? • Can my child get enough vitamin D from sources other than supplements? • Will my baby receive vitamin D through breast milk? • Can my baby get enough vitamin D from going outside? • What kinds of affordable meals can I prepare which contain vitamin D?	
*Importance of vitamin D*	
• Why is vitamin D important? • What are the health benefits of vitamin D? • What are the benefits of taking vitamin D supplements for me and my child? • Why do we need vitamin D in our diets? • What does vitamin D do to your body? • Why is vitamin D important to take during pregnancy and breastfeeding? • What are the risks of not getting enough vitamin D in the diet? • What happens if you take too little or too much vitamin D? • How much sunlight is needed to get enough vitamin D?	
*Vitamin D supplements for my baby/child*	
• Why do I need to give my baby/child a vitamin D supplement? • When do I need to give a vitamin D supplement to my baby/child? • Does my baby need vitamin D drops from birth when breastfeeding? • Does my baby/child need vitamin D supplements when drinking formula milk? • Does my baby/child need vitamin D supplements when drinking cow’s milk? • How much vitamin D does my baby/child need? • How much vitamin D does my baby need when breastfeeding? • How long do I need to give a vitamin D supplement to my baby/child for? • How often should I give a vitamin D supplement to my baby/child? • Which vitamin D supplement should I give to my baby/child? • How do I give vitamin D supplements to my baby/child? E.g. during weaning? • Are vitamin D supplements the only/best option for my baby?	
*Vitamin D supplements when breastfeeding or pregnant*	
• How much vitamin D do I need as a breastfeeding mother? • How long should I take vitamin D for when breastfeeding? • How often should I take a vitamin D supplement when pregnant and breastfeeding? • Why do I need to take vitamin D when breastfeeding? • Why does my milk not give my baby enough vitamin D?	
*Accessing vitamin D supplements*	
• Where can we get vitamin D supplements from? • Which vitamin D supplement should I be taking when breastfeeding? • Which vitamin D supplements should I use?	

#### Delivery of vitamin D information

The questionnaire asked *‘how would you want this information (vitamin D information) to be delivered?* (Respondents could choose more than one option*).* Of those that said they would like more information, 71% (*n* = 118), out of 166 respondents to the question, 28 non-response) said they would want a chat with a health visitor, and 65% (*n* = 108) said a chat with a midwife. 20% (*n* = 33) wanted a chat with a GP. Examples provided within the qualitative findings included: during routine appointments, baby weigh in clinics, antenatal classes, health visitor clinics, breastfeeding visits, provided during weaning and during a child’s routine appointments at the GPs e.g. vaccinations.

*“I think probably verbally is maybe better, or maybe a mix of the two. If someone tells you about it, you’ve got it on your mind and then you’ve got a backup when you see information in writing as well” (parent, interview 2)*

Furthermore, 57% (*n* = 95, out of 166 respondents) of parents surveyed wanted a leaflet or booklet. Only 13% (*n* = 21) of parents would want the information delivered through an Application on a mobile device (App). However, other suggestions included 28% (*n* = 47) via email, 27% (*n* = 44) through a website, and 11% (*n* = 19) via text message*.* Other suggestions from the qualitative findings included: an online “messenger” service with a health professional, and through YouTube videos, with information videos on healthy eating appropriate for children.

*“…there are Apps and websites you can sign up to about your baby that tell you what you should be doing at certain points…maybe if it was in something like that you would take notice of it because it is a weekly update and it is not too much information in one go” (parent, interview 3)*

The qualitative findings also revealed that parents wanted smaller bits of information, rather than a lot of information at once. Some thought the information could go within the breastfeeding information, others would like it to be provided with healthy diet information and should be more tailored to individual feeding practices, e.g. breastfeeding or formula feeding. Parents wanted to receive the information more frequently throughout pregnancy and after the birth of the baby. Furthermore, many thought that the information needed to be repeated regularly, e.g. by different health professionals for consolidation or with reminders about vitamin D intake.

*“I do think if it were drip-fed, you were given things about feeding, things about vitamin D separately in separate meetings, you have time to read it and digest it. Whereas if it is just given to you, some people just put it in their bag and won’t look at it again” (parent, interview 4)*

*“If you just touch on it a little bit (during pregnancy) and then later on, bring the information back when it is a bit more useful for your child” (parent, interview 3)*

*“it might be reminders linked in with the check-ups, is she still eating her vitamin D? If she’s not, have you made sure she’s eating x, y, z to get this amount of vitamin D she should be having? And then you’ve got in in your head” (parent, interview 1)*

#### Presentation of information

Parents wanted more specific and clearer vitamin D information, with simpler and easier to interpret written information. Some thought that the information needs to be more visible and eye catching, with better advertising e.g. in supermarkets, schools, children’s centres and places people visit every day.

*“Make it look like it is important, stand out and say this is important, it needs to be promoted as well as folic acid is” (parent 1, focus group 4)*

#### Health messages about vitamin D

Focus group/interview participants (*N* = 18), were asked the following questions relating to the types of health messages they would like to see in the vitamin D information and to discuss which factors would be most likely to encourage them to increase their vitamin D intake*:*

*“If we think about what messages we would like to see about vitamin D, what would be more helpful?*: *a message that scared you (e.g. a young mother whose baby was born with rickets because she did not consume enough vitamin D during her pregnancy), or a positive message (e.g. adequate vitamin D can help you build strong bones and may prevent certain types of cancer)”*.

Parents expressed mixed views. Some parents preferred the idea of a more positive message about the health effects of vitamin D, rather than something outlining the risks of not taking vitamin D.

*“I am not sure how much scare tactics work, might make you panic more. I think by positively reinforcing the message would be the best way” (parent, interview 2)*

Others however, thought a message that “*scared*” them might be more effective in encouraging behaviour change.

*“People react more to it when they learn about the potential harm caused to baby” (parent 1, focus group 3)*

Whilst others thought that both a more positive message and a risk message about the health effects of vitamin D would encourage more people to increase their vitamin D intake.

*“Because you have got the extreme of not taking it but you have also got the proactive approach of the positive outcome of taking it, so you know why you are taking it…both makes it more black and white rather than a huge grey area in the middle” (parent 2, focus group 4)*

When asked what type of message would most likely influence their behaviour, the majority of parents thought that the message should focus on both the immediate benefits and the long-term benefits of vitamin D.

*“Parents want to know about the health effects on their children now and in the future” (parent 3, focus group 4)*

The participants were then asked,

“*Behaviours are often difficult to change, because they are so ingrained in our daily routines. What do you think we could do that would get you to make changes to your child’s diet? E.g. Eating/drinking more foods containing vitamin D or taking vitamin D supplements?”*

The following suggestions were made: promotions from GPs or health visitors e.g. give out free vitamin D drops/ supplements or a free product; and improved parent knowledge about vitamin D e.g. health visitor goes through information leaflets clearly and clearly explains the benefits; parents role modelling healthy behaviours e.g. families eating together at meal times and all eating the same food; better publicity about vitamin D e.g. adverts on television, promotions “something easier to see”; reminders about vitamin D; and clear explanations on how to give your child vitamin D supplements and ideas on how to incorporate vitamin D into a daily routine e.g. mix with food or liquids.

*“…having that knowledge makes you want to do the best for your child and being explicit about that, like talking through leaflets and understanding the benefits. Because they did it with breastfeeding and most of my friends who were breastfeeding could reel off the benefits, but you don’t ever get the same with vitamin D” (parent, interview 4)*

Parents were asked *“overall what health messages relating to vitamin D would be most relevant to you? E.g. maintenance of healthy bones and teeth, reduced risk of flu and colds, and healthy pregnancy”.* The majority of parents stated that they would like to see all the health messages presented in the education around vitamin D, especially information about strong bones and teeth and the Department of Health recommendations.

### Understanding and awareness of vitamin D

#### Awareness of the importance of vitamin D for the body

The parents were then asked in the questionnaire ‘*why is vitamin D important for the body?*’ (respondents could choose more than one option). The results revealed that 23% (*n* = 45, out of 194 respondents) of parents were not sure why vitamin D is important for the body, 64% (*n* = 125) were able to say that it was important for strong bones and teeth and 39% (*n* = 76) identified that it can strengthen the immune system, however only 13% (*n* = 26) were aware that vitamin D can help retain eyesight.

#### Awareness of recommendations around vitamin D intake

In order to further explore parents’ awareness and understanding around vitamin D recommendations, parents were asked during the focus group/interviews (*N* = 18), if they were aware of the recommendations for vitamin D during pregnancy and for their child.

The findings revealed that some parents were aware of the need for vitamin D supplementation during pregnancy (either because their midwife had informed them or via their own research), however several parents reported that they were not aware of the recommendations; there was more knowledge of the need for folic acid supplementation than vitamin D. Moreover, many parents were not aware of the recommendations for giving their baby/child a vitamin D supplement and some parents reported confusion over current recommendations regarding breastfeeding and vitamin D supplement use for mother and infant. Furthermore, some reported uncertainty over how to give vitamin D supplements to their baby when exclusively breastfed.

*“The health visitor didn’t clearly explain at home visits. A lot more information needed around weaning as despite looking I still don’t feel confident that I have the right information regarding what should I take and she receive through breastmilk or does she need drops straight away or should she be getting it by going outside?” (parent, questionnaire feedback)*

*“I think the information changed once my baby was born and we were a bit confused. I was breastfeeding and taking a vitamin D supplement and the new recommendations, the advice was that my baby should also have it, because it wasn’t enough and then it was, is it going to be too much? Do I still take it? Is it just them? Which one is best for them? How do we give it, juice? So all of this was quite confusing” (parent, interview 4)*

*“No, I was not aware of those recommendations, the only thing I knew was to take my little one to catch some sun” (written interview response)*

When asked whether they knew about the recommended dose to be taken during pregnancy, only a few could recall the dose of vitamin D required from the nutrition label on their multivitamin mainly. Moreover, only a few parents knew what dose of vitamin D was recommended for their baby/child. When asked if they thought that most pregnant women and parents would follow these recommendations for vitamin D supplementation, a few parents thought that they would, because parents in their own circle of friends reported providing vitamin D supplements for their children. Whereas, many others perceived that there would be a general lack of awareness because pregnant women and parents are not being adequately informed about the need for vitamin D supplements by their health practitioners. Moreover, some perceived that if parents were told about the need for vitamin D supplementation during pregnancy and for their baby/child, they would follow the recommendations out of concern for the health of their baby

*“Parents are not well informed enough about the importance of vitamin D to give their children vitamin D supplements” (parent 1, focus group 4)*

Focus group/interview participants (*N* = 18) were also asked if they had been informed about the Healthy Start Scheme and where they could obtain vitamin D supplements. Only some parents reported that their midwife or health visitor had mentioned the scheme and where to access free supplements. Others had not been specifically told, but had read about it or heard about it from other information sources.

#### Frequency of use of vitamin D supplements

**Fig. 1:**
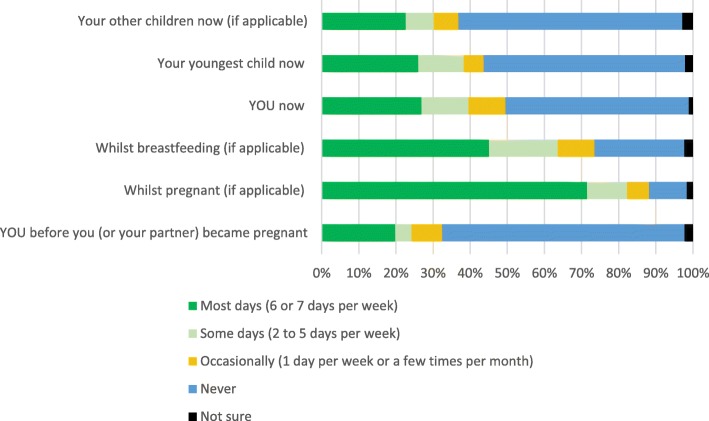
How often do you and/or your child(ren) take a Vitamin D supplement, or a multivitamin containing Vitamin D?

Current guidelines state that infants receiving more than 500 ml of infant formula do not need a vitamin D supplement. Of 193 respondents (1 non-response), 31%, (*n* = 60) reported that their child was receiving infant formula of which 60% (*n* = 36) were receiving more than 500 ml a day. Meaning under the current Public Health England or Department of Health recommendations, 158 respondents out of the total sample of 194, should be giving their child a vitamin D supplement. Figure 1 indicates that only 26%, *n* = 49 (out of 192 respondents to the question, 2 non-response) of parents were giving their youngest child a vitamin D supplement on most days of the week. 69% (*n* = 133) of the sample took a vitamin D supplement during pregnancy, but this decreased to 41% (*n* = 78) when breastfeeding.

When asked why they were giving their children a vitamin D supplement (respondents could choose more than one option), 34% (*n* = 33, out of 97 respondents to the question, 97 non response) of parents reported because of a health visitor recommendation and 29% (*n* = 28) stated because it was part of a multi-vitamin. 20% (*n* = 19) reported because of recommended national guidelines, 13% (*n* = 13) stated to improve health, 10% (*n* = 10) stated recommended by other health professional and 3% (*n* = 3) said other.

#### Vitamin D fortified foods and drinks

##### Which fortified foods and drinks respondents have bought

The questionnaire asked *‘Have you ever bought any food or drink products because they have been fortified with vitamin D?* ‘Out of 193 respondents to the question (1 non-response), only 21% (*n* = 41) of parents reported that they had bought foods because they have been fortified with vitamin D.

##### Willingness to buy vitamin D fortified foods/drinks

The questionnaire then asked ‘*from the list of options which food and drink products would you be willing to buy to increase your child’s vitamin D intake?*’ (respondents could choose more than one option). 90% (*n* = 169, out of 188 respondents to the question, 6 non response) said yogurts or yogurt drinks, 79% (*n* = 148) said breakfast cereals, 62% (*n* = 117) cheese, 57% bread (*n* = 108), 62% (*n* = 116) fortified toddler and baby foods (excluding infant formula), 59% (*n* = 111) milk or milk-based drinks (excluding infant formula), 42% (*n* = 79) fruit juice, 38% (*n* = 71) infant formula and 33% (*n* = 62) margarine.

##### Facilitators and barriers to purchasing vitamin D fortified products

Parents were then asked *‘What would encourage you to buy foods and drinks fortified with vitamin D?’* (respondents could choose more than one option). 70% (*n* = 133, of 191 respondents to the question, 3 non-response) thought that having more information about the link between vitamin D and health might encourage them to buy foods and drinks fortified with vitamin D; as well as information about which foods and drinks contain vitamin D (62%, *n* = 118) and 62% (*n* = 118) also wanted suitable products for babies and children. Around a quarter of respondents (27%, *n* = 52) also wanted a healthy product, better availability in local shops and supermarkets (26%, *n* = 50), lower costs (23%, *n* = 44) and tasty (23%, *n* = 44). Additional analysis found no significant differences by respondent’s age, ethnicity, highest qualification, deprivation and child’s age. Focus group/interview participants (*N* = 18), also added the following suggestions: clearer labelling of vitamin D content and benefits of vitamin D, products specifically lower in sugar and salt, more offers and promotions on products and better advertising.

Focus group/interview participants (*N* = 18), revealed that the main barriers to purchasing vitamin D fortified products included: a lack of awareness of which products are available; insufficient labelling about vitamin D content on products and how it meets child’s daily requirements; fear of insufficient vitamin D intake from food, when children do not consume all their food; poor availability of products suitable for babies/toddlers; healthiness of product e.g. high sugar content; price; possibility of overdosing, e.g. supplement use with fortified foods; may not see the need to buy fortified products; habitual buying of the same products each time and other factors are considered of more importance than vitamin D content.

*“If they were advertised in a way where you could clearly see that they had vitamin D in. I think the problem with fortified foods in general is it is hard to trail through the back of the product and it can be really quite small on the packaging. If it was a bit clearer on the packaging that would be good, it would save a bit of time” (parent, interview 4)*

Parents taking part in a focus group/interview were asked if they would like more information about fortified foods and drinks within the information provided about vitamin D. The majority of parents said that they would like more information about fortified foods and drinks. Some said that this information could be presented in an information leaflet, for example a list of fortified foods and drinks could be added to the lists of suitable foods provided during pregnancy. When asked specifically what information they would like to know about fortified products, the following suggestions were provided: the benefits of vitamin D in drinks/foods and the importance of vitamin D; the consequences of not getting enough vitamin D in the diet, how the quantity of vitamin D in fortified products meets the Recommended Daily Intake for a child, e.g. as a percentage; and safety of consuming fortified foods and drinks for fear of overdosing on vitamin D.

*“I think a little more, not necessarily to scare me, but the consequences of not having it. It’s alright saying they need it, but why? Maybe if I know a bit more detail as to why they need it, why it is so important, because that might increase the urgency of me taking it or making sure she gets it” (parent, interview 1)*

*“Obviously you don’t want to get three products that all have vitamin D in them and find out you are having too much” (parent 1, focus group 4)*

##### Preferred ways to increase vitamin D intake

Parents in the focus group/interviews (*N* = 18), were also asked what their preferred methods would be to increase vitamin D intake both during pregnancy and for their child. Many said that a vitamin D supplement would be preferential during pregnancy. The main reasons for this included; a supplement is quick and easy, food preferences can change during pregnancy and many pregnant mothers eat far less; it is also not clear how much vitamin D you are getting from food.

*“It is easier to take a supplement everyday rather than having to think about the right food choices” (parent, interview 3)*

*“it would be good to know that I am getting absolutely everything I needed from my diet, but you don’t always know how much is in the foods you are eating, so if you are getting enough. I think it feels more reassured that you know what you are taking, this specific dose every day, so you are getting exactly what you need” (parent, interview 2)*

A few others said they would prefer to obtain vitamin D from “healthy food choices” and sunlight. Some parents preferred the idea of giving their child vitamin D drops or a supplement to achieve their child’s required vitamin D intake. The following reasons were given; a supplement is easier, and it is difficult to give a child appropriate foods and drinks when they are fussy eaters.

Others however, would prefer to give their child “healthy foods” containing vitamin D or for them to “play outside”, as some struggled to give their child a supplement because they did not like the taste.

*“If he was getting it all from his diet, that would be better” (parent, interview 2)*

Furthermore, a few parents said that they would be happy to give their child a yogurt fortified with vitamin D for example, if it was clear how it would meet their child’s recommended daily intake, as this would be easier than having to remember a supplement.

*“The yogurt, because I wouldn’t have to worry about, I’m just thinking about when she sleeps over and she goes to places, yogurt would be easier and I know she likes yogurt, she eats yogurt” (parent, interview 1)*

## Discussion

The results from this study revealed that a significant proportion of parents wanted more information about vitamin D intake at key stages from pregnancy until the child is 2 years old. Only around half of parents reported receiving information about vitamin D from their midwife or health visitor. Overall, the lack of awareness around the importance of vitamin D intake for children is a concern that is highlighted in this study and supports previous research [3, 25, 30–32]. The results also highlighted that parents felt that the information was of poor quality. Promotion of the guidelines for vitamin D intake and the timing of this information, needs to be improved to be more effective in changing vitamin D intake in children aged 0 to 2 years of age. Furthermore, it needs to be considered a daily habit [31, 33, 34].

The results from previous research are very similar to the current findings with parents of children under 5 years unaware that their child is at risk of vitamin D deficiency and parents often reporting that they had never received information about the need for vitamin D [13, 25]. Our findings emulate previous research conducted in 2010 concluding that the situation was unchanged and parents remain unfamiliar with the government recommendations around supplementation, during pregnancy, breastfeeding and weaning stages [30]. This sample was a well-educated population, which makes the findings of more concern that awareness and understanding of the importance of vitamin D intake in children in this sample is poor, and therefore highly likely that awareness would be lower in those less educated, which could result in further health inequalities. Another study with participants who on average had high education levels was conducted in Ireland, and indicated that whilst there was awareness of the need for vitamin D in the diet, daily supplementation was low [31]. Suggesting not only does there need to be an improvement in public awareness of the importance of vitamin D and the issue of vitamin D deficiency, but also to improve parents understanding of dietary sources of vitamin D including products fortified with vitamin D.

Several parents reported that they were unaware of the recommendations for vitamin D at key time points, e.g. during pregnancy, breastfeeding, formula milk intake and at the age of 1 year – when cow’s milk is introduced and there is a decrease in formula milk intake. Some thought that the recommendations around breastfeeding and vitamin D supplement use for mother and child were limited and unclear. Others could not access information about vitamin D from a suitable health care practitioner, or could not remember it amongst the vast amount of information given and did not find the presentation of information appealing, which suggests a wider public health campaign is needed [34, 35]. Parents are aware of the need for folic acid during pregnancy, but few knew about vitamin D requirements [12]. This issue is consistent with previous research [31, 36], indicating that the recommendations for vitamin D supplementation relating to breastfeeding and during infancy need to be made much clearer both within the written information and discussed regularly by both midwives and health visitors [12]. This study identified that nearly two thirds of parents reported that they would consult a medical professional for information about health and nutrition (61%). This suggests that vitamin D information would be well received by parents if it was provided by their general practitioner, at times such as during immunisation visits.

A high percentage of parents (69%) reported that they took a vitamin D supplement on most days of the week during pregnancy. Yet, only 26% of the sample reported giving their youngest child a vitamin D supplement on most days of the week. It is worth noting that 34% of those who reported giving their youngest child vitamin D, did so because they were advised to by their health visitor and 20% because they were aware of the national guidelines. Demonstrating the important role health visitors are having in providing this information to parents at local level [25, 34].

There were very mixed results in whether parents felt they would use fortified foods for vitamin D intake. Less than a quarter of parents had bought foods/drinks because they had been fortified with vitamin D. Overall willingness to purchase certain products fortified with vitamin D was however, high. Research conducted in Ireland found that fortified foods make up a significant contribution to vitamin D intake without risk of excessively high levels [37]. Fortified milk was considered an ideal option to improve vitamin D intake [37]. Combined with a Government public health campaign, fortified milk would be a suitable way to improve vitamin intake from the age of 1 year and onwards. It could also be easily implemented nationwide in older children (age 3 years) as they are providing free milk at nurseries across England [37]. However, the key to encouraging parents of young children to buy food and drinks fortified with vitamin D was again linking education around the importance of preventing vitamin deficiency and which products were fortified in vitamin D clearly labelled. Further research is needed to determine if consumption of fortified foods only, rather than supplementation, means children actually meet their daily intake of vitamin D [37].

### Strengths and limitations of the study

The strength of this study is that it uses a combination of quantitative and qualitative methods, with a high response rate of 194. The data from the questionnaire identifies a range of factors and this is supported by the in-depth and detailed responses from the focus groups and interviews with parents.

Whilst, our sample was representative in terms of ethnic diversity of the Leeds population and the population nationally, (87% White British, compared to the Leeds average of 85% and 86% national average), the sample was not however, representative of the Leeds population in terms of socioeconomic characteristics. Only 10% of the sample were from the most 20% deprived areas, compared to an average of 31% for Leeds and 20% nationally [29]. A much larger proportion of the sample had a level 4 qualification (69%), than the Leeds average (34%), or the national average (38%) [29]. Therefore, the results lack some representation from communities of lower socioeconomic status and varying ethnic backgrounds. Furthermore, it was a self-selected sample, therefore the parents are more likely to be aware of, or interested in vitamin D.

Therefore, the results lack some representation from communities of a lower socioeconomic status and varying ethnic backgrounds. In order to overcome this in future work, establishing key contacts working with diverse communities (for example in the council’s public health team) could be beneficial.

### Recommendations for improving information about vitamin D

- Vitamin D information should be delivered in the first instance during routine appointments, through discussions with a midwife and health visitor with a supportive information leaflet provided. Midwives and health visitors should be provided with up to date guidance about vitamin D recommendations.
- This information should be delivered regularly during routine appointments at timely stages throughout pregnancy, and after the birth, instead of altogether.
- Information could be delivered during antenatal classes, baby weigh in clinics, breastfeeding/midwife support worker visits and GP consultations or during child vaccinations and during weaning.
- Other useful sources for vitamin D information could include: sign-posting to approved websites (e.g. hospital website, medical websites, parenting websites), emails, texts, smartphone Apps, an online messenger service with a health professional responding, and YouTube videos.
- Reminders about vitamin D supplementation for pregnant women and for parents (e.g. verbal reminders, email alerts, texts) may be useful and parents should be given an option to sign up for them.
- All pregnant mothers and parents need to be informed about the Healthy Start Scheme and where to obtain vitamin D supplements. There could be further promotion about the scheme at children’s centres and nurseries for example.

### Presentation and content of vitamin D advice/information

- Needs to be simpler, easier to read with more specific and clearer guidelines about vitamin D. It also needs to be more eye catching and appealing in appearance.
- The guidelines relating to vitamin D intake for mother and child during breastfeeding specifically needs to be made simpler and clearer. This information could accompany the information about nutrition during pregnancy or breastfeeding.

### The vitamin D information needs to attempt to address as many of the following themes

- Where we get vitamin D from.
- The importance of vitamin D: information about how vitamin D relates to health, including information about strong bones and teeth and the Department of Health Vitamin D recommendations. It could also contain a positive and a risk message about the health effects of vitamin D and the immediate and long term benefits of vitamin D.
- Vitamin D supplements and access to vitamin D supplements for my baby/child.
- Taking vitamin D supplements when breastfeeding or pregnant.
- Vitamin D rich food and drinks.
- Types of foods and drinks fortified with vitamin D.
- How to incorporate vitamin D into a daily routine, with clear instructions on how to give a baby or child vitamin D supplementation.

### Recommendations for commercial organisations

- In order to encourage people to buy fortified products, there needs to be clearer labelling on the importance/benefits of vitamin D and availability of vitamin D from a product e.g. how quantity of vitamin D meets a child’s recommended daily intake.
- Information about which foods and drinks are fortified with vitamin D, and about the link between vitamin D and health.
- Products need to be suitable for babies and toddlers, lower costs, with healthy options available; with lower sugar and salt content, tasty, longer shelf life and better availability in local shops and supermarkets.
- Improved advertising of fortified products.

## Conclusion

The findings from this study indicated that around half of the parents reported receiving no information about vitamin D during pregnancy, breastfeeding or for their child. The majority obtained information by proactively seeking information via a range of medical and parenting websites. There was evidence that the recommendations for vitamin D were not being followed by pregnant or breastfeeding women or parents, as levels of vitamin D supplementation were lower than they should be. Furthermore, many parents reported a lack of awareness or confusion over the current guidelines around vitamin D supplementation (including the Healthy Start Scheme) in babies from birth, particularly in relation to breastfeeding, but also afterwards including dietary sources and the potential role of vitamin D fortified products. Vitamin D intake needs to be included in routine health checks. The willingness of parents to purchase products fortified with vitamin D suggests a potential role for these products to contribute to increasing the intake of vitamin D in children. There needs to be a national level campaign to successfully change the current practices.

## Additional file

Additional file 1 Vitamin D and your child. Questionnaire developed for this research study (DOCX 37 kb)

## Data Availability

The study data can be obtained from the corresponding author on reasonable request.

## Abbreviations

App: Application on a mobile device
DH: Department of Health
EYP: Early Years Practitioner
GP: General Practitioner (family doctor)
HND: Higher National Diploma
IMD: Index of Multiple Deprivation
IRAS: Integrated Research Application System
NDNS: National Diet and Nutrition Survey
NHS: National Health Service
NICE: National Institute for Health and Care Excellence
PHE: Public Health England
SACN: Scientific Advisory Committee on Nutrition
UK: United Kingdom
UVB: Ultraviolet B sunlight
