# Supplementary material for: We still don’t know that our children need vitamin D daily: a study of parents’ understanding of vitamin D requirements in children aged 0-2 years
Source: BMC Public Health. 2019 Aug 15;19:1119. doi: 10.1186/s12889-019-7340-x (PMC6694627; doi:10.1186/s12889-019-7340-x)
Supplement: Supplementary file 1 — Vitamin D and your child. Questionnaire developed for this research study (DOCX 37 kb) [file 12889_2019_7340_MOESM1_ESM.docx]

**Vitamin D and your child**

The aim of this survey is to find out how we can improve vitamin D in the diets of pregnant women and children under 2 years of age.

**Please only complete this survey if you are responsible for a child under 2 years old.**

**If you have two children under the age of 2, please think about your youngest child when answering the questions.**

This survey is split into 4 sections and will take about 5-10 minutes to complete.

The survey will run from 16^th^ February to the 31st May 2017. Respondents will be entered into a prize draw to win a £50 Mothercare voucher. The winner will be contacted by email before the 1^st^ June 2017. Please provide your email address on the last page of the survey if you wish to be entered into the prize draw.

The survey is anonymous and responses are strictly confidential. We will not share or publish any information that could lead to the identification of individuals.

The research is being carried out by Leeds Beckett University and is being funded by Nutricia Early Life Nutrition in association with the Royal National Orthopaedic Hospital NHS Trust. If you have any queries please email vitaminD@leedsbeckett.ac.uk

**Section 1 – Information about vitamin D**

**1. Where do you generally go for information and advice on health and nutrition?**

*(Please tick all that apply)*

| ❑ | Medical websites e.g. NHS choices | ❑ | Health professionals |
| --- | --- | --- | --- |
| ❑ | Parenting websites e.g. Netmums | ❑ | Friends and family |
| ❑ | Health and fitness websites | ❑ | Information leaflets or booklets |
| ❑ | Online community forums | ❑ | Books |
| ❑ | Blogs | ❑ | Magazines |
| ❑ | Youtube | ❑ | Other, please specify__________________ |
| ❑ | I have not looked for health and nutrition information before |  |  |
|  |  | | |

**2. Have you ever searched for information on vitamin D?** *(Please tick all that apply)*

| ❑ | Yes, relating to Vitamin D and pregnancy |  |
| --- | --- | --- |
| ❑ | Yes, relating to Vitamin D and breastfeeding |  |
| ❑ | Yes, relating to Vitamin D for my child |  |
| ❑ | No |  |

**3. Did you (or your partner) receive information about vitamin D from any of the following sources?** *(Please answer all options)*

| **During 1 to 1 sessions** | **Yes during pregnancy** | **Yes after birth** | **No** | **Not applicable** |
| --- | --- | --- | --- | --- |
| Your midwife | ❑ | ❑ | ❑ | ❑ |
| Your health visitor | ❑ | ❑ | ❑ | ❑ |
| Your GP | ❑ | ❑ | ❑ | ❑ |
| Breastfeeding support worker | ❑ | ❑ | ❑ | ❑ |
| Early years practitioner  e.g. children’s centre worker, child minder, private nursery staff | ❑ | ❑ | ❑ | ❑ |
| **During group sessions and other** |  |  |  |  |
| Group or class run by healthcare professionals and/or children’s centre | ❑ | ❑ | ❑ | ❑ |
| Group or class run by a private company | ❑ | ❑ | ❑ | ❑ |
| NHS email or text messaging service | ❑ | ❑ | ❑ | ❑ |
| Other source, please specify  ____________________________ | ❑ | ❑ | ❑ | ❑ |

**If you did NOT receive any information about vitamin D, please go to question 7**

**4. How did you receive information on vitamin D? (Please tick all that apply)**

| ❑ | A leaflet or booklet |
| --- | --- |
| ❑ | A discussion |
| ❑ | Signposted to a website |
| ❑ | Other please specify_____________________________________________ |

**5. How would you rate the information you received about vitamin D?**

| ❑ | Very good |
| --- | --- |
| ❑ | Good |
| ❑ | OK |
| ❑ | Poor |
| ❑ | Very poor |

**6. How could the information you received about vitamin D be improved?** (e.g. type of information, timing, quantity, quality)

**7. Would you have liked more information about vitamin D?** *(Please tick all that apply)*

| ❑ | Yes about vitamin D in pregnancy |
| --- | --- |
| ❑ | Yes about vitamin D and breastfeeding |
| ❑ | Yes about vitamin D for my child |
| ❑ | No |
| ❑ | Not sure |

**8. If yes, how would you like this information?**

| ❑ | A chat with my midwife | ❑ | Website |
| --- | --- | --- | --- |
| ❑ | A chat with my health visitor | ❑ | App |
| ❑ | A chat with my GP | ❑ | Email |
| ❑ | From a children’s centre | ❑ | Text |
| ❑ | A group session | ❑ | Leaflet or booklet |
|  |  | ❑ | Other, please specify____________________ |

**Section 2 - Your understanding of vitamin D**

**9.** **What are the best food sources of vitamin D? (Please tick all that apply)**

| ❑ | Eggs |
| --- | --- |
| ❑ | Green leafy vegetables |
| ❑ | Red meat |
| ❑ | Oily fish |
| ❑ | Carrots |
| ❑ | Not sure |

**10. Why is vitamin D important for the body? (Please tick all that apply)**

| ❑ | Strong bones and teeth |
| --- | --- |
| ❑ | Eyesight |
| ❑ | Liver function |
| ❑ | Strengthening the immune system |
| ❑ | Not sure |

**11. How would you rate your understanding of vitamin D?**

|  | Very good | Good | Ok | Poor | Very poor |
| --- | --- | --- | --- | --- | --- |
| Before pregnancy | ❑ | ❑ | ❑ | ❑ | ❑ |
| Now | ❑ | ❑ | ❑ | ❑ | ❑ |

**Section 3 – Vitamin D in your and your family’s diet**

Certain food and drinks have vitamin D added to them and are called **fortified foods and drinks**

**12. Have you ever bought any food or drink products BECAUSE they have been fortified with vitamin D?**

| ❑ | Yes, please specify which products______________________________________ |
| --- | --- |
| ❑ | No |

**13. What would encourage you to buy foods and drinks fortified with vitamin D? (Please tick all that apply)**

| ❑ | More information about which food or drink products that have been fortified with vitamin D |
| --- | --- |
| ❑ | More information about the link between vitamin D and health |
| ❑ | Suitable for babies and young children |
| ❑ | Lower cost |
| ❑ | Better availability in local shops and supermarkets |
| ❑ | Longer shelf life |
| ❑ | Tasty |
| ❑ | Healthy product |
| ❑ | Other (please specify) ______________________________________ |

**14. Which food and drink products would you be willing to buy to increase your child’s vitamin D intake?**

|  | **Yes** | **No** |
| --- | --- | --- |
| Fortified yoghurts or yoghurt drinks | ❑ | ❑ |
| Fortified cheese | ❑ | ❑ |
| Fortified milk or milk based drinks *(excluding infant formula)* | ❑ | ❑ |
| Fortified margarine | ❑ | ❑ |
| Fortified fruit juice | ❑ | ❑ |
| Fortified bread | ❑ | ❑ |
| Fortified breakfast cereals | ❑ | ❑ |
| Fortified baby and toddler foods *(excluding infant formula)* *e.g. biscuits, baby food* | ❑ | ❑ |
| Infant formula (all types) | ❑ | ❑ |
| Other, please specify____________­________________ | ❑ | ❑ |

**15. Does your child currently receive infant formula (any type)?**

| ❑ | Yes |
| --- | --- |
| ❑ | No |

**16. If yes, how much do they receive in a 24 hour period? A large bottle = 280mls**

**(9 fl oz) and a small bottle = 150mls (5 fl oz)**

| ❑ | Less than 500ml (17 fl oz) |
| --- | --- |
| ❑ | More than 500ml (17 fl oz) |
| ❑ | Not sure |

**17. How often do you and/or your child(ren) take a vitamin D supplement, or a multivitamin containing vitamin D?**

|  | **Most days**  (6 -7 days per week) | **Some days**  (2 -5 days per week) | **Occasionally** (1 day per week or a few times per month) | **Never** | **Not sure** |
| --- | --- | --- | --- | --- | --- |
| **YOU** before you (or your partner) became pregnant | ❑ | ❑ | ❑ | ❑ | ❑ |
| Whilst pregnant (if applicable) | ❑ | ❑ | ❑ | ❑ | ❑ |
| Whilst breastfeeding (if applicable) | ❑ | ❑ | ❑ | ❑ | ❑ |
| **YOU** now | ❑ | ❑ | ❑ | ❑ | ❑ |
| **Your youngest child** now | ❑ | ❑ | ❑ | ❑ | ❑ |
| **Your other children** now (if applicable) | ❑ | ❑ | ❑ | ❑ | ❑ |

**18. If you and/or your child currently take a vitamin D supplement, what are the reasons for this?** *(Please tick all that apply)*

|  | **YOU** |  | **YOUR CHILD** |  |
| --- | --- | --- | --- | --- |
| ❑ | Recommended by health visitor | ❑ | Recommended by health visitor |  |
| ❑ | Recommended by midwife | ❑ | Recommended by midwife |  |
| ❑ | Recommended by other health professional | ❑ | Recommended by other health professional |  |
| ❑ | Recommended national guidelines | ❑ | Recommended national guidelines |  |
| ❑ | To improve health | ❑ | To improve health |  |
| ❑ | Part of a multivitamin | ❑ | Part of a multivitamin |  |
| ❑ | Other, please specify__________________ | ❑ | Other, please specify____________________ |  |

**20. Where do you get your vitamin D supplements from?**

|  | **For yourself** | **For your child** |
| --- | --- | --- |
| Free from children’s centre | ❑ | ❑ |
| Buy them from children’s centre | ❑ | ❑ |
| From a pharmacy on prescription | ❑ | ❑ |
| Buy them from a pharmacy, health food shop, supermarket | ❑ | ❑ |

**Section 4 - About you and your family**

| **21. Are you?** | | | |  |  |
| --- | --- | --- | --- | --- | --- |
| ❑ | Mum | ❑ | Dad | ❑ | Other e.g. grandparent, foster carer. Please specify_________ |

**22. Please provide your youngest child’s age in months ____________**

**23. How old are you?**

| ❑ | Under 18 | ❑ | 25 - 29 | ❑ | 35 - 39 | ❑ | 45 or over |
| --- | --- | --- | --- | --- | --- | --- | --- |
| ❑ | 18 - 24 | ❑ | 30 - 34 | ❑ | 40 - 44 |  |  |

**24. What is your highest qualification?**

| ❑ | **No qualifications** |
| --- | --- |
| ❑ | **Level 1** (e.g. fewer than 5 GCSEs at grades A-C, foundation GNVQ, NVQ 1,  intermediate 1 national qualification (Scotland) or equivalent) |
| ❑ | **Level 2** (e.g. 5 or more GCSEs at grades A-C, intermediate GNVQ, NVQ 2,  intermediate 2 national qualification (Scotland) or equivalent) |
| ❑ | **Level 3** (e.g. 2 or more A levels, advanced GNVQ, NVQ 3,  2 or more higher or advanced higher national qualifications (Scotland) or equivalent) |
| ❑ | **Level 4** (e.g. HND, Degree and Higher Degree level qualifications or equivalent) |
| ❑ | **Other**, please specify____________________ |

**25. Are you currently taking part in the Healthy Start Programme, which provides free vouchers to buy healthy foods or drinks, infant formula and vitamins?**

| ❑ | Yes |
| --- | --- |
| ❑ | No |
| ❑ | Not sure |

**26. Which country were you born in?**

| ❑ | UK - England, Wales, Scotland or Northern Ireland |
| --- | --- |
| ❑ | Other country, please specify_______________________ |
|  | When did you first start living in the UK? ______________ |

**27. What is your ethnic group?**

|  | **A White** |  | **C Asian/ Asian British** |
| --- | --- | --- | --- |
| ❑ | English/Welsh/Scottish/Northern Irish/British | ❑ | Indian |
| ❑ | Irish | ❑ | Pakistani |
| ❑ | Gypsy or Irish Traveller | ❑ | Bangladeshi |
| ❑ | Any other white background | ❑ | Chinese |
|  | **B Mixed/ Multiple ethnic groups** | ❑ | Any other Asian background |
| ❑ | White and Black Caribbean |  | **D Black/ African/ Caribbean background** |
| ❑ | White and Black African | ❑ | African |
| ❑ | White and Asian | ❑ | Caribbean |
| ❑ | Any other mixed / multiple ethnic background | ❑ | Any other Black/African/Caribbean background |
|  |  |  | **E Other ethnic group** |
|  |  | ❑ | Arab |
|  |  | ❑ | Any other ethnic group  *(please specify)____________________* |

**28. What is your postcode? ________________**

**29. Is there anything else you would like to add?**

If you would like to be entered into the prize draw, please provide your email address below. Partially completed surveys will not be entered into the prize draw.

**Email:** __________ __________

Please leave blank if you do not want to be entered into the prize draw. We will not share your email address with third parties.

We are currently looking for pregnant women and parents with children under 2 years of age to take part in a 30 minute focus group in the Leeds/Bradford area. You will receive a £5 high street gift voucher for taking part. If you would like further information about this, please tick the box below

❑ Please send me further information about the focus groups

**Thank you for completing the survey**
